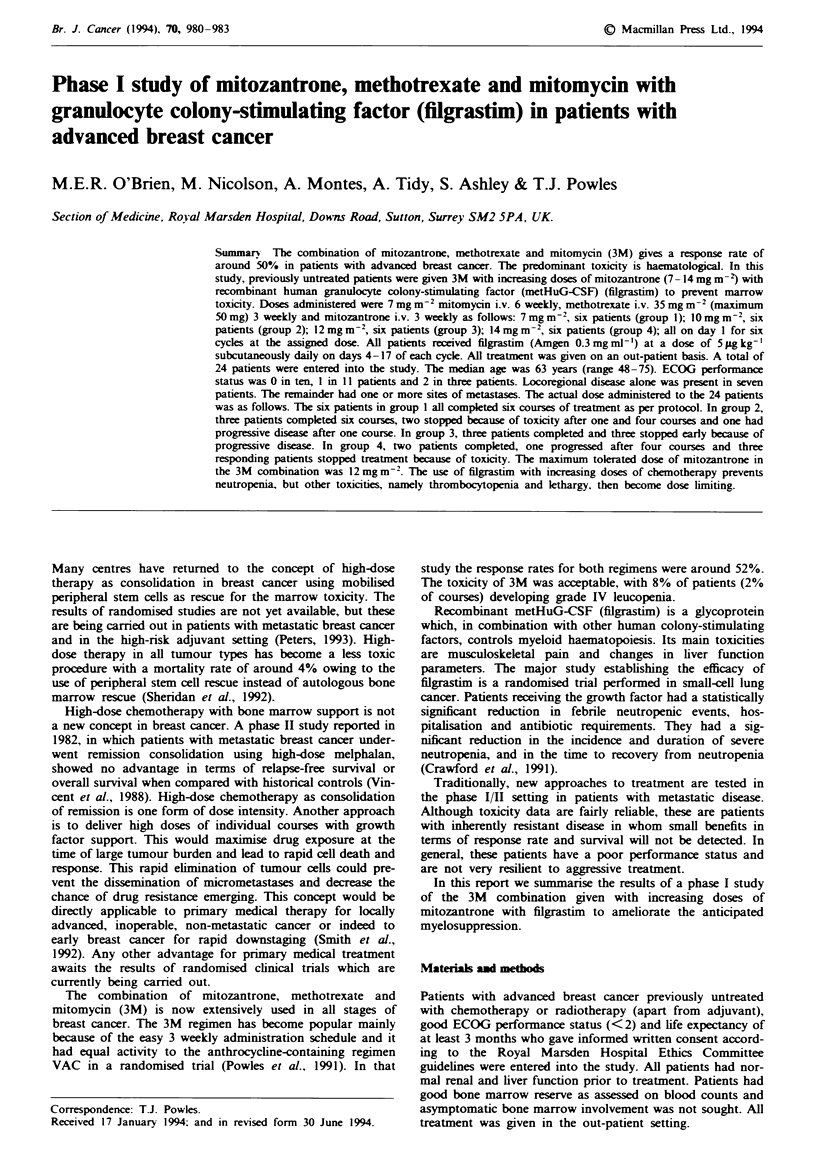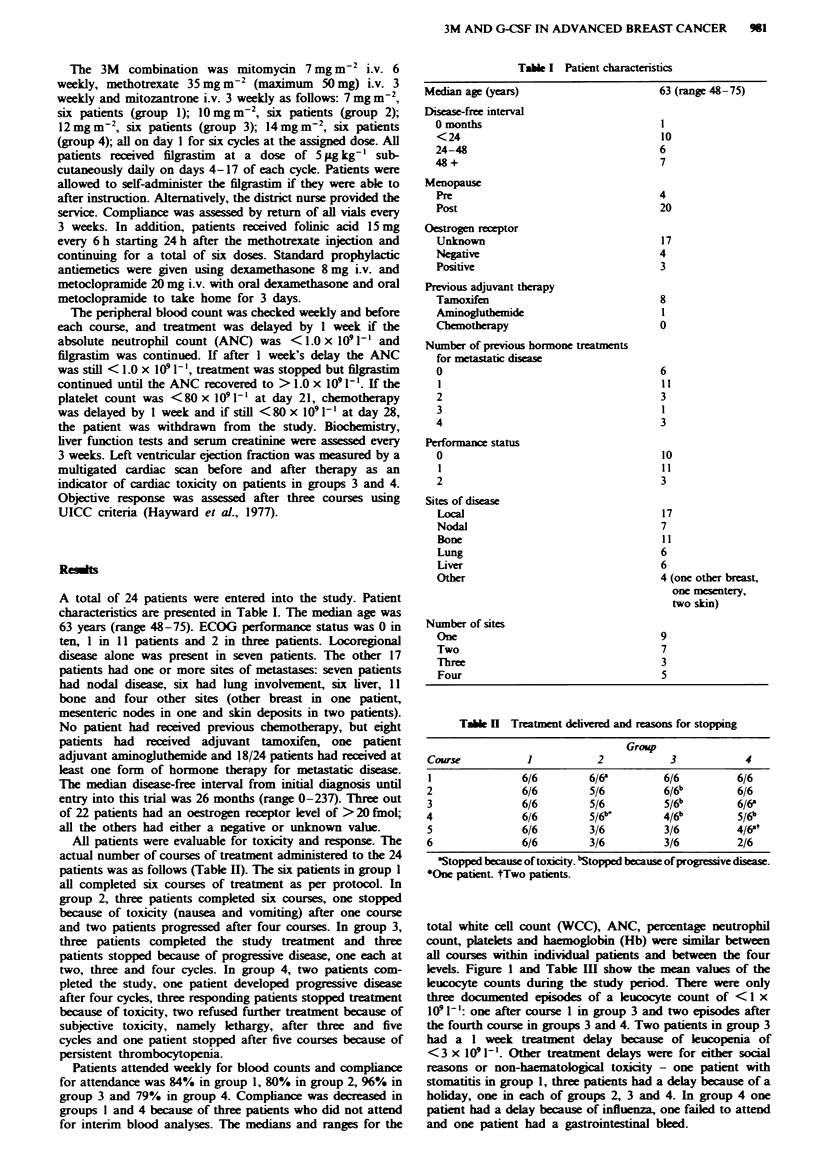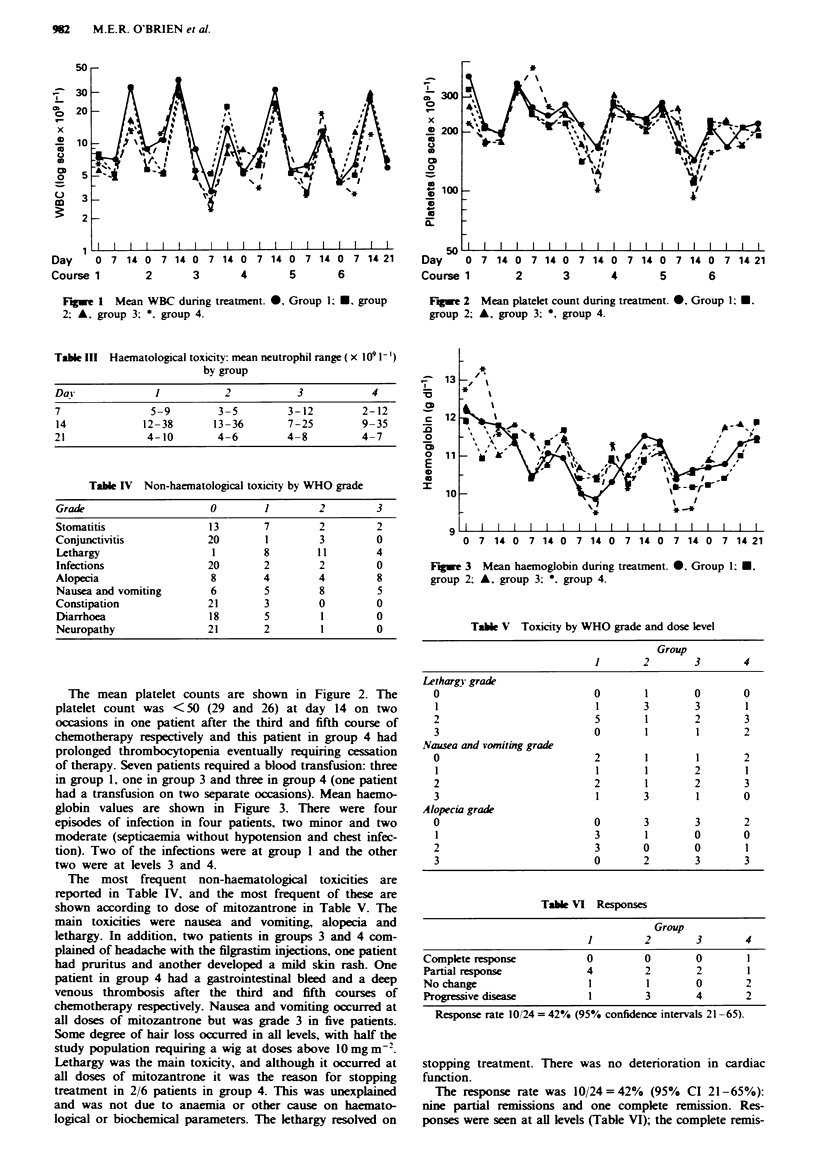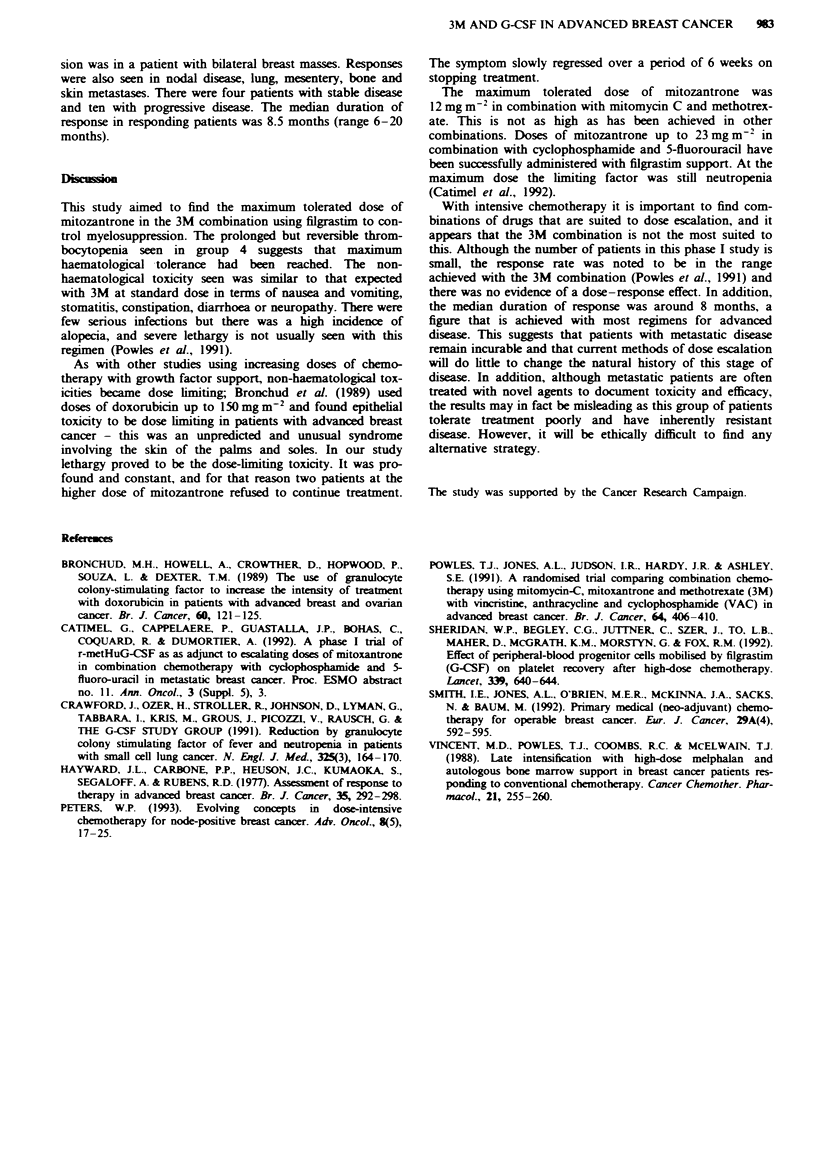# Phase I study of mitozantrone, methotrexate and mitomycin with granulocyte colony-stimulating factor (filgrastim) in patients with advanced breast cancer.

**DOI:** 10.1038/bjc.1994.433

**Published:** 1994-11

**Authors:** M. E. O'Brien, M. Nicolson, A. Montes, A. Tidy, S. Ashley, T. J. Powles

**Affiliations:** Section of Medicine, Royal Marsden Hospital, Sutton, Surrey, UK.

## Abstract

The combination of mitozantrone, methotrexate and mitomycin (3M) gives a response rate of around 50% in patients with advanced breast cancer. The predominant toxicity is haematological. In this study, previously untreated patients were given 3M with increasing doses of mitozantrone (7-14 mg m-2) with recombinant human granulocyte colony-stimulating factor (metHuG-CSF) (filgrastim) to prevent marrow toxicity. Doses administered were 7 mg m-2 mitomycin i.v. 6 weekly, methotrexate i.v. 35 mg m-2 (maximum 50 mg) 3 weekly and mitozantrone i.v. 3 weekly as follows: 7 mg m-2, six patients (group 1); 10 mg m-2, six patients (group 2); 12 mg m-2, six patients (group 3); 14 mg m-2, six patients (group 4); all on day 1 for six cycles at the assigned dose. All patients received filgrastim (Amgen 0.3 mg ml-1) at a dose of 5 micrograms kg-1 subcutaneously daily on days 4-17 of each cycle. All treatment was given on an out-patient basis. A total of 24 patients were entered into the study. The median age was 63 years (range 48-75). ECOG performance status was 0 in ten, 1 in 11 patients and 2 in three patients. Locoregional disease alone was present in seven patients. The remainder had one or more sites of metastases. The actual dose administered to the 24 patients was as follows. The six patients in group 1 all completed six courses of treatment as per protocol. In group 2, three patients completed six courses, two stopped because of toxicity after one and four courses and one had progressive disease after one course. In group 3, three patients completed and three stopped early because of progressive disease. In group 4, two patients completed, one progressed after four courses and three responding patients stopped treatment because of toxicity. The maximum tolerated dose of mitozantrone in the 3M combination was 12 mg m-2. The use of filgrastim with increasing doses of chemotherapy prevents neutropenia, but other toxicities, namely thrombocytopenia and lethargy, then become dose limiting.


© Macmillan Press Ltd., 1994

### Phase I study of mitozantrone, methotrexate and mitomycin with granulocyte colony-stimulating factor (filgrastim) in patients with advanced breast cancer

M.E.R. O'Brien, M. Nicolson, A. Montes, A. Tidy, S. Ashley & T.J. Powles

*Section of Medicine, Royal Marsden Hospital, Downs Road, Sutton, Surrey SM2 5PA, UK.*

**Summary** The combination of mitozantrone, methotrexate and mitomycin (3M) gives a response rate of around 50% in patients with advanced breast cancer. The predominant toxicity is haematological. In this study, previously untreated patients were given 3M with increasing doses of mitozantrone (7-14 mg $m^{-2}$) with recombinant human granulocyte colony-stimulating factor (metHuG-CSF) (filgrastim) to prevent marrow toxicity. Doses administered were 7 mg $m^{-2}$ mitomycin i.v. 6 weekly, methotrexate i.v. 35 mg $m^{-2}$ (maximum 50 mg) 3 weekly and mitozantrone i.v. 3 weekly as follows: 7 mg $m^{-2}$, six patients (group 1); 10 mg $m^{-2}$, six patients (group 2); 12 mg $m^{-2}$, six patients (group 3); 14 mg $m^{-2}$, six patients (group 4); all on day 1 for six cycles at the assigned dose. All patients received filgrastim (Amgen 0.3 mg $ml^{-1}$) at a dose of 5 $\mu g$ $kg^{-1}$ subcutaneously daily on days 4-17 of each cycle. All treatment was given on an out-patient basis. A total of 24 patients were entered into the study. The median age was 63 years (range 48-75). ECOG performance status was 0 in ten, 1 in 11 patients and 2 in three patients. Locoregional disease alone was present in seven patients. The remainder had one or more sites of metastases. The actual dose administered to the 24 patients was as follows. The six patients in group 1 all completed six courses of treatment as per protocol. In group 2, three patients completed six courses, two stopped because of toxicity after one and four courses and one had progressive disease after one course. In group 3, three patients completed and three stopped early because of progressive disease. In group 4, two patients completed, one progressed after four courses and three responding patients stopped treatment because of toxicity. The maximum tolerated dose of mitozantrone in the 3M combination was 12 mg $m^{-2}$. The use of filgrastim with increasing doses of chemotherapy prevents neutropenia, but other toxicities, namely thrombocytopenia and lethargy, then become dose limiting.

Many centres have returned to the concept of high-dose therapy as consolidation in breast cancer using mobilised peripheral stem cells as rescue for the marrow toxicity. The results of randomised studies are not yet available, but these are being carried out in patients with metastatic breast cancer and in the high-risk adjuvant setting (Peters, 1993). High-dose therapy in all tumour types has become a less toxic procedure with a mortality rate of around 4% owing to the use of peripheral stem cell rescue instead of autologous bone marrow rescue (Sheridan *et al.*, 1992).

High-dose chemotherapy with bone marrow support is not a new concept in breast cancer. A phase II study reported in 1982, in which patients with metastatic breast cancer underwent remission consolidation using high-dose melphalan, showed no advantage in terms of relapse-free survival or overall survival when compared with historical controls (Vincent *et al.*, 1988). High-dose chemotherapy as consolidation of remission is one form of dose intensity. Another approach is to deliver high doses of individual courses with growth factor support. This would maximise drug exposure at the time of large tumour burden and lead to rapid cell death and response. This rapid elimination of tumour cells could prevent the dissemination of micrometastases and decrease the chance of drug resistance emerging. This concept would be directly applicable to primary medical therapy for locally advanced, inoperable, non-metastatic cancer or indeed to early breast cancer for rapid downstaging (Smith *et al.*, 1992). Any other advantage for primary medical treatment awaits the results of randomised clinical trials which are currently being carried out.

The combination of mitozantrone, methotrexate and mitomycin (3M) is now extensively used in all stages of breast cancer. The 3M regimen has become popular mainly because of the easy 3 weekly administration schedule and it had equal activity to the anthrocycline-containing regimen VAC in a randomised trial (Powles *et al.*, 1991). In that study the response rates for both regimens were around 52%. The toxicity of 3M was acceptable, with 8% of patients (2% of courses) developing grade IV leucopenia.

Recombinant metHuG-CSF (filgrastim) is a glycoprotein which, in combination with other human colony-stimulating factors, controls myeloid haematopoiesis. Its main toxicities are musculoskeletal pain and changes in liver function parameters. The major study establishing the efficacy of filgrastim is a randomised trial performed in small-cell lung cancer. Patients receiving the growth factor had a statistically significant reduction in febrile neutropenic events, hospitalisation and antibiotic requirements. They had a significant reduction in the incidence and duration of severe neutropenia, and in the time to recovery from neutropenia (Crawford *et al.*, 1991).

Traditionally, new approaches to treatment are tested in the phase I/II setting in patients with metastatic disease. Although toxicity data are fairly reliable, these are patients with inherently resistant disease in whom small benefits in terms of response rate and survival will not be detected. In general, these patients have a poor performance status and are not very resilient to aggressive treatment.

In this report we summarise the results of a phase I study of the 3M combination given with increasing doses of mitozantrone with filgrastim to ameliorate the anticipated myelosuppression.

### Materials and methods

Patients with advanced breast cancer previously untreated with chemotherapy or radiotherapy (apart from adjuvant), good ECOG performance status (<2) and life expectancy of at least 3 months who gave informed written consent according to the Royal Marsden Hospital Ethics Committee guidelines were entered into the study. All patients had normal renal and liver function prior to treatment. Patients had good bone marrow reserve as assessed on blood counts and asymptomatic bone marrow involvement was not sought. All treatment was given in the out-patient setting.

Correspondence: T.J. Powles.

Received 17 January 1994; and in revised form 30 June 1994.

The 3M combination was mitomycin 7 mg m$^{-2}$ i.v. 6 weekly, methotrexate 35 mg m$^{-2}$ (maximum 50 mg) i.v. 3 weekly and mitozantrone i.v. 3 weekly as follows: 7 mg m$^{-2}$, six patients (group 1); 10 mg m$^{-2}$, six patients (group 2); 12 mg m$^{-2}$, six patients (group 3); 14 mg m$^{-2}$, six patients (group 4); all on day 1 for six cycles at the assigned dose. All patients received filgrastim at a dose of 5 $\mu$g kg$^{-1}$ subcutaneously daily on days 4–17 of each cycle. Patients were allowed to self-administer the filgrastim if they were able to after instruction. Alternatively, the district nurse provided the service. Compliance was assessed by return of all vials every 3 weeks. In addition, patients received folinic acid 15 mg every 6 h starting 24 h after the methotrexate injection and continuing for a total of six doses. Standard prophylactic antiemetics were given using dexamethasone 8 mg i.v. and metoclopramide 20 mg i.v. with oral dexamethasone and oral metoclopramide to take home for 3 days.

The peripheral blood count was checked weekly and before each course, and treatment was delayed by 1 week if the absolute neutrophil count (ANC) was $<1.0 \times 10^9$ l$^{-1}$ and filgrastim was continued. If after 1 week's delay the ANC was still $<1.0 \times 10^9$ l$^{-1}$, treatment was stopped but filgrastim continued until the ANC recovered to $>1.0 \times 10^9$ l$^{-1}$. If the platelet count was $<80 \times 10^9$ l$^{-1}$ at day 21, chemotherapy was delayed by 1 week and if still $<80 \times 10^9$ l$^{-1}$ at day 28, the patient was withdrawn from the study. Biochemistry, liver function tests and serum creatinine were assessed every 3 weeks. Left ventricular ejection fraction was measured by a multigated cardiac scan before and after therapy as an indicator of cardiac toxicity on patients in groups 3 and 4. Objective response was assessed after three courses using UICC criteria (Hayward *et al.*, 1977).

### Results

A total of 24 patients were entered into the study. Patient characteristics are presented in Table I. The median age was 63 years (range 48–75). ECOG performance status was 0 in ten, 1 in 11 patients and 2 in three patients. Locoregional disease alone was present in seven patients. The other 17 patients had one or more sites of metastases: seven patients had nodal disease, six had lung involvement, six liver, 11 bone and four other sites (other breast in one patient, mesenteric nodes in one and skin deposits in two patients). No patient had received previous chemotherapy, but eight patients had received adjuvant tamoxifen, one patient adjuvant aminogluthemide and 18/24 patients had received at least one form of hormone therapy for metastatic disease. The median disease-free interval from initial diagnosis until entry into this trial was 26 months (range 0–237). Three out of 22 patients had an oestrogen receptor level of >20 fmol; all the others had either a negative or unknown value.

All patients were evaluable for toxicity and response. The actual number of courses of treatment administered to the 24 patients was as follows (Table II). The six patients in group 1 all completed six courses of treatment as per protocol. In group 2, three patients completed six courses, one stopped because of toxicity (nausea and vomiting) after one course and two patients progressed after four courses. In group 3, three patients completed the study treatment and three patients stopped because of progressive disease, one each at two, three and four cycles. In group 4, two patients completed the study, one patient developed progressive disease after four cycles, three responding patients stopped treatment because of toxicity, two refused further treatment because of subjective toxicity, namely lethargy, after three and five cycles and one patient stopped after five courses because of persistent thrombocytopenia.

Patients attended weekly for blood counts and compliance for attendance was 84% in group 1, 80% in group 2, 96% in group 3 and 79% in group 4. Compliance was decreased in groups 1 and 4 because of three patients who did not attend for interim blood analyses. The medians and ranges for the total white cell count (WCC), ANC, percentage neutrophil count, platelets and haemoglobin (Hb) were similar between all courses within individual patients and between the four levels. Figure 1 and Table III show the mean values of the leucocyte counts during the study period. There were only three documented episodes of a leucocyte count of $<1 \times 10^9$ l$^{-1}$: one after course 1 in group 3 and two episodes after the fourth course in groups 3 and 4. Two patients in group 3 had a 1 week treatment delay because of leucopenia of $<3 \times 10^9$ l$^{-1}$. Other treatment delays were for either social reasons or non-haematological toxicity – one patient with stomatitis in group 1, three patients had a delay because of a holiday, one in each of groups 2, 3 and 4. In group 4 one patient had a delay because of influenza, one failed to attend and one patient had a gastrointestinal bleed.

**Table I** Patient characteristics

| | |
|---|---|
| Median age (years) | 63 (range 48–75) |
| Disease-free interval | |
| 0 months | 1 |
| <24 | 10 |
| 24–48 | 6 |
| 48 + | 7 |
| Menopause | |
| Pre | 4 |
| Post | 20 |
| Oestrogen receptor | |
| Unknown | 17 |
| Negative | 4 |
| Positive | 3 |
| Previous adjuvant therapy | |
| Tamoxifen | 8 |
| Aminogluthemide | 1 |
| Chemotherapy | 0 |
| Number of previous hormone treatments for metastatic disease | |
| 0 | 6 |
| 1 | 11 |
| 2 | 3 |
| 3 | 1 |
| 4 | 3 |
| Performance status | |
| 0 | 10 |
| 1 | 11 |
| 2 | 3 |
| Sites of disease | |
| Local | 17 |
| Nodal | 7 |
| Bone | 11 |
| Lung | 6 |
| Liver | 6 |
| Other | 4 (one other breast, one mesentery, two skin) |
| Number of sites | |
| One | 9 |
| Two | 7 |
| Three | 3 |
| Four | 5 |

**Table II** Treatment delivered and reasons for stopping

| | Group | | | |
|---|---|---|---|---|
| *Course* | *1* | *2* | *3* | *4* |
| 1 | 6/6 | 6/6[a] | 6/6 | 6/6 |
| 2 | 6/6 | 5/6 | 6/6[b] | 6/6 |
| 3 | 6/6 | 5/6 | 5/6[b] | 6/6[a] |
| 4 | 6/6 | 5/6[b*] | 4/6[b] | 5/6[b] |
| 5 | 6/6 | 3/6 | 3/6 | 4/6[a†] |
| 6 | 6/6 | 3/6 | 3/6 | 2/6 |

[a]Stopped because of toxicity. [b]Stopped because of progressive disease. *One patient. †Two patients.

**Figure 1** Mean WBC during treatment. ●, Group 1; ■, group 2; ▲, group 3; *, group 4.

**Figure 2** Mean platelet count during treatment. ●, Group 1; ■, group 2; ▲, group 3; *, group 4.

**Table III** Haematological toxicity: mean neutrophil range ( × $10^9$ $l^{-1}$) by group

| *Day* | *1* | *2* | *3* | *4* |
|---|---|---|---|---|
| 7 | 5–9 | 3–5 | 3–12 | 2–12 |
| 14 | 12–38 | 13–36 | 7–25 | 9–35 |
| 21 | 4–10 | 4–6 | 4–8 | 4–7 |

**Table IV** Non-haematological toxicity by WHO grade

| *Grade* | *0* | *1* | *2* | *3* |
|---|---|---|---|---|
| Stomatitis | 13 | 7 | 2 | 2 |
| Conjunctivitis | 20 | 1 | 3 | 0 |
| Lethargy | 1 | 8 | 11 | 4 |
| Infections | 20 | 2 | 2 | 0 |
| Alopecia | 8 | 4 | 4 | 8 |
| Nausea and vomiting | 6 | 5 | 8 | 5 |
| Constipation | 21 | 3 | 0 | 0 |
| Diarrhoea | 18 | 5 | 1 | 0 |
| Neuropathy | 21 | 2 | 1 | 0 |

The mean platelet counts are shown in Figure 2. The platelet count was <50 (29 and 26) at day 14 on two occasions in one patient after the third and fifth course of chemotherapy respectively and this patient in group 4 had prolonged thrombocytopenia eventually requiring cessation of therapy. Seven patients required a blood transfusion: three in group 1, one in group 3 and three in group 4 (one patient had a transfusion on two separate occasions). Mean haemoglobin values are shown in Figure 3. There were four episodes of infection in four patients, two minor and two moderate (septicaemia without hypotension and chest infection). Two of the infections were at group 1 and the other two were at levels 3 and 4.

The most frequent non-haematological toxicities are reported in Table IV, and the most frequent of these are shown according to dose of mitozantrone in Table V. The main toxicities were nausea and vomiting, alopecia and lethargy. In addition, two patients in groups 3 and 4 complained of headache with the filgrastim injections, one patient had pruritus and another developed a mild skin rash. One patient in group 4 had a gastrointestinal bleed and a deep venous thrombosis after the third and fifth courses of chemotherapy respectively. Nausea and vomiting occurred at all doses of mitozantrone but was grade 3 in five patients. Some degree of hair loss occurred in all levels, with half the study population requiring a wig at doses above 10 mg $m^{-2}$. Lethargy was the main toxicity, and although it occurred at all doses of mitozantrone it was the reason for stopping treatment in 2/6 patients in group 4. This was unexplained and was not due to anaemia or other cause on haematological or biochemical parameters. The lethargy resolved on stopping treatment. There was no deterioration in cardiac function.

**Figure 3** Mean haemoglobin during treatment. ●, Group 1; ■, group 2; ▲, group 3; *, group 4.

**Table V** Toxicity by WHO grade and dose level

| | Group | | | |
|---|---|---|---|---|
| | *1* | *2* | *3* | *4* |
| *Lethargy grade* | | | | |
| 0 | 0 | 1 | 0 | 0 |
| 1 | 1 | 3 | 3 | 1 |
| 2 | 5 | 1 | 2 | 3 |
| 3 | 0 | 1 | 1 | 2 |
| *Nausea and vomiting grade* | | | | |
| 0 | 2 | 1 | 1 | 2 |
| 1 | 1 | 1 | 2 | 1 |
| 2 | 2 | 1 | 2 | 3 |
| 3 | 1 | 3 | 1 | 0 |
| *Alopecia grade* | | | | |
| 0 | 0 | 3 | 3 | 2 |
| 1 | 3 | 1 | 0 | 0 |
| 2 | 3 | 0 | 0 | 1 |
| 3 | 0 | 2 | 3 | 3 |

**Table VI** Responses

| | Group | | | |
|---|---|---|---|---|
| | *1* | *2* | *3* | *4* |
| Complete response | 0 | 0 | 0 | 1 |
| Partial response | 4 | 2 | 2 | 1 |
| No change | 1 | 1 | 0 | 2 |
| Progressive disease | 1 | 3 | 4 | 2 |

Response rate 10/24 = 42% (95% confidence intervals 21–65).

The response rate was 10/24 = 42% (95% CI 21–65%): nine partial remissions and one complete remission. Responses were seen at all levels (Table VI); the complete remission was in a patient with bilateral breast masses. Responses were also seen in nodal disease, lung, mesentery, bone and skin metastases. There were four patients with stable disease and ten with progressive disease. The median duration of response in responding patients was 8.5 months (range 6–20 months).

### Discussion

This study aimed to find the maximum tolerated dose of mitozantrone in the 3M combination using filgrastim to control myelosuppression. The prolonged but reversible thrombocytopenia seen in group 4 suggests that maximum haematological tolerance had been reached. The non-haematological toxicity seen was similar to that expected with 3M at standard dose in terms of nausea and vomiting, stomatitis, constipation, diarrhoea or neuropathy. There were few serious infections but there was a high incidence of alopecia, and severe lethargy is not usually seen with this regimen (Powles *et al.*, 1991).

As with other studies using increasing doses of chemotherapy with growth factor support, non-haematological toxicities became dose limiting; Bronchud *et al.* (1989) used doses of doxorubicin up to 150 mg $m^{-2}$ and found epithelial toxicity to be dose limiting in patients with advanced breast cancer – this was an unpredicted and unusual syndrome involving the skin of the palms and soles. In our study lethargy proved to be the dose-limiting toxicity. It was profound and constant, and for that reason two patients at the higher dose of mitozantrone refused to continue treatment. The symptom slowly regressed over a period of 6 weeks on stopping treatment.

The maximum tolerated dose of mitozantrone was 12 mg $m^{-2}$ in combination with mitomycin C and methotrexate. This is not as high as has been achieved in other combinations. Doses of mitozantrone up to 23 mg $m^{-2}$ in combination with cyclophosphamide and 5-fluorouracil have been successfully administered with filgrastim support. At the maximum dose the limiting factor was still neutropenia (Catimel *et al.*, 1992).

With intensive chemotherapy it is important to find combinations of drugs that are suited to dose escalation, and it appears that the 3M combination is not the most suited to this. Although the number of patients in this phase I study is small, the response rate was noted to be in the range achieved with the 3M combination (Powles *et al.*, 1991) and there was no evidence of a dose–response effect. In addition, the median duration of response was around 8 months, a figure that is achieved with most regimens for advanced disease. This suggests that patients with metastatic disease remain incurable and that current methods of dose escalation will do little to change the natural history of this stage of disease. In addition, although metastatic patients are often treated with novel agents to document toxicity and efficacy, the results may in fact be misleading as this group of patients tolerate treatment poorly and have inherently resistant disease. However, it will be ethically difficult to find any alternative strategy.

The study was supported by the Cancer Research Campaign.

### References

BRONCHUD, M.H., HOWELL, A., CROWTHER, D., HOPWOOD, P., SOUZA, L. & DEXTER, T.M. (1989) The use of granulocyte colony-stimulating factor to increase the intensity of treatment with doxorubicin in patients with advanced breast and ovarian cancer. *Br. J. Cancer*, **60**, 121–125.

CATIMEL, G., CAPPELAERE, P., GUASTALLA, J.P., BOHAS, C., COQUARD, R. & DUMORTIER, A. (1992). A phase I trial of r-metHuG-CSF as as adjunct to escalating doses of mitoxantrone in combination chemotherapy with cyclophosphamide and 5-fluoro-uracil in metastatic breast cancer. Proc. ESMO abstract no. 11. *Ann. Oncol.*, **3** (Suppl. 5), 3.

CRAWFORD, J., OZER, H., STROLLER, R., JOHNSON, D., LYMAN, G., TABBARA, I., KRIS, M., GROUS, J., PICOZZI, V., RAUSCH, G. & THE G-CSF STUDY GROUP (1991). Reduction by granulocyte colony stimulating factor of fever and neutropenia in patients with small cell lung cancer. *N. Engl. J. Med.*, **325**(3), 164–170.

HAYWARD, J.L., CARBONE, P.P., HEUSON, J.C., KUMAOKA, S., SEGALOFF, A. & RUBENS, R.D. (1977). Assessment of response to therapy in advanced breast cancer. *Br. J. Cancer*, **35**, 292–298.

PETERS, W.P. (1993). Evolving concepts in dose-intensive chemotherapy for node-positive breast cancer. *Adv. Oncol.*, **8**(5), 17–25.

POWLES, T.J., JONES, A.L., JUDSON, I.R., HARDY, J.R. & ASHLEY, S.E. (1991). A randomised trial comparing combination chemotherapy using mitomycin-C, mitoxantrone and methotrexate (3M) with vincristine, anthracycline and cyclophosphamide (VAC) in advanced breast cancer. *Br. J. Cancer*, **64**, 406–410.

SHERIDAN, W.P., BEGLEY, C.G., JUTTNER, C., SZER, J., TO, L.B., MAHER, D., MCGRATH, K.M., MORSTYN, G. & FOX, R.M. (1992). Effect of peripheral-blood progenitor cells mobilised by filgrastim (G-CSF) on platelet recovery after high-dose chemotherapy. *Lancet*, **339**, 640–644.

SMITH, I.E., JONES, A.L., O'BRIEN, M.E.R., MCKINNA, J.A., SACKS, N. & BAUM, M. (1992). Primary medical (neo-adjuvant) chemotherapy for operable breast cancer. *Eur. J. Cancer*, **29A**(4), 592–595.

VINCENT, M.D., POWLES, T.J., COOMBS, R.C. & MCELWAIN, T.J. (1988). Late intensification with high-dose melphalan and autologous bone marrow support in breast cancer patients responding to conventional chemotherapy. *Cancer Chemother. Pharmacol.*, **21**, 255–260.